# Perspectives of Protocol Based Breaking Bad News among Medical Patients and Physicians in a Teaching Hospital, Ethiopia

**DOI:** 10.4314/ejhs.v30i6.21

**Published:** 2020-11

**Authors:** Henok Fisseha, Wudneh Mulugeta, Rodas A Kassu, Temesgen Geleta, Hailemichael Desalegn

**Affiliations:** 1 Department of Internal medicine, St. Paul's Hospital Millennium Medical College, Addis Ababa, Ethiopia; 2 Harvard Medical School, Department of Medicine-Cambridge Health Alliance; 3 Department of Neurosurgery, St. Paul's Hospital Millennium Medical College, Addis Ababa, Ethiopia; 4 Department of Public Health, St. Paul's Hospital Millennium Medical College, Addis Ababa, Ethiopia

**Keywords:** breaking bad news, SPIKES protocol, medical patients, satisfaction

## Abstract

**Background:**

Discussing potentially bad outcomes is a standard communication task in clinical care. Physicians' awareness on ways to communicate bad news is considered low. SPIKES protocol is the most popular strategy used by physicians, but its practice and patients' perception are not known. This study attempted to fill the knowledge gap on protocol implementation, patient preference and physician effects.

**Methods:**

Hospital-based descriptive cross-sectional study was conducted at SPHMMC from May 1 to June 30 using structured interviews administered to patients and physicians. Three hundred and sixty patients and 111 physicians were included. Assessment of SPIKES performance, patient satisfaction, patient preference, and physician awareness, attitude and effects were studied.

**Results:**

Performance of SPIKES protocol was setting (74.5%), perception (51.1%), invitation (56.3%), knowledge (15.9%), emotion (22.3%) and summary (10.1%). Only 30.6% of the patients were entirely satisfied with the interaction, and 19.2% with knowledge attained. Patient satisfaction was associated with physician asking how much information they like (P=0.025). Patient desire and report showed variation. Eighty-two percent of the physicians were not aware of the protocol, and 83.8% had no training. Half of the physicians feel depressed after disclosure.

**Conclusions:**

Patient satisfaction with communication process and knowledge is poor, as is performance of SPIKES components. Satisfaction is related to being asked how much patients want to know. Patients' desires on how to be told news is different from how it is done. Breaking bad news increases feeling of depression. Awareness and training on the protocol are deficient; medical schools should incorporate it into their studies and implement proper follow-up.

## Introduction

Discussing serious news or breaking “bad news” (BBN) is a standard communication task in clinical care (1). “Bad news” has been defined as “any information likely to alter a patient's view of his/her future drastically…” (2).

Good patient-centered communication is associated with better adherence to treatment, pain control, satisfaction, emotional health, functional status, trust and physical outcomes (1, 3). If not communicated the right way, it can result in impaired emotional response on patients as well as, excessive stress, negative attitudes toward treating staff, miscommunication and poor outcome (1,4,5,6,7). There is variation in what specific information patients want to know (1). Studies show that patients are not satisfied with ways physicians communicate news (4,5). BBN can negatively impact the clinician resulting in stress, difficulty handling emotions, job dissatisfaction, poor rapport and litigation (1,8,9).

One of the most popular and accepted strategies to BBN worldwide is the SPIKES (Setting up, Perception, Invitation, Knowledge, Emotions, Strategy and Summary) protocol published in 2000 by Walter F. Baile. It has gained immense popularity; is used in all range of communications; in western countries has achieved guideline status; and incorporated in many curriculums as part of communication skills training (10,11). The name is an acronym describing steps of a conversation. Starting with S, preparing for the talk, through P, and I—where physician determines the patient's perception and readiness to receive news; which precede information breakout, —K. E, responding to the patient's emotions follows. Eventually, S follows (4,10,11). It has a particular application for cancer patients but has been used in fields other than oncology including Human Immunodeficiency Virus, patients on chronic dialysis, spinal cord injury, and physicians working in almost all areas (2,12,13,14,15,16,17,18).

Level of training was less than desired in South Korean and Canadian physician studies and worse in most of less developed countries including Iran, Turkey, India and Pakistan (8,16,18,19). In a 2013 Korean study, 80% of trainees followed the protocol compared to none of participating physicians being aware of any protocol in another (16,17).

There is a widespread problem with physicians' and students' skills and knowledge on standardized ways of BBN. Improvements are needed on knowledge, attitude, skill and practice of using SPIKES (4,17,18,19). Data regarding knowledge and utilization of such protocols, patient desires regarding components of the protocol and satisfaction has not been studied in Ethiopia and in developing countries as a whole.

This study will determine the performance of the protocol, satisfaction with BBN to medical patients, and their preference; assess current knowledge, attitude, and effect on physicians, and help recommend ways to improve them. To the researchers' knowledge, this will be the first study done on this topic on medical patients in Ethiopia. It will have significant implications including improving patient care and satisfaction and increase physicians' awareness and practice of the protocol and serve as an evidence-based input to improving medical curriculum design.

The specific objectives of this study were to measure the implementation of SPIKES protocol when delivering bad news, patient preference, and associated satisfaction and to determine physician knowledge, attitude, and its effects.

## Subjects and Methods

**Study area, period and design**: The study was conducted in St. Paul's Hospital Millennium Medical College (SPHMMC), a referral and teaching hospital located in Addis Ababa, Ethiopia (20). The patients admitted to a medical ward or following in outpatient clinics between May 1 to June 30, 2019, with a diagnosis fitting the criteria of bad news as well as all physicians working in the Internal Medicine Department of the college served as the target population. A descriptive cross-sectional study was conducted. The study population included patients admitted to the medical ward and patients visiting outpatient clinics in the specified period who received perceived bad news about disease and physicians working in the wards during the time of the interview.

**Sample size and sampling method**: The sample size was calculated using a single population proportion formula taking a prevalence of 50 percent since the performance of SPIKES protocol is not known, confidence level of 95% and the margin of error of 5% was used. The total number of patients expected to be admitted in the study period was 208, and 6,820 patients were expected to be seen in the referral clinics of the department, according to hospital registry, giving a total patient of 7,028. A final sample size of 364 patients was obtained.

Convenience sampling method was used, and all patients fulfilling the inclusion criteria were included until the required sample size was met.

Among the 156 physicians working in the department, using a similar formula, 111 physicians were included and stratified as 26 seniors, 61 residents, eight general practitioners, and 16 interns selected using convenience sampling.

**Data collection method and tool**: Primary data was obtained by administering a structured interview prepared by reviewing different kinds of literature and undertaking modifications for the population studied to patients.

The level of performance of SPIKES was evaluated by calculating percentage of the average of the sum of responses to specific questions. Components of SPIKES were assessed in 12 questions answered on a 4 part Likert scale.

A self-administered questionnaire containing 32 questions was provided to physicians. The inclusion criteria included all patients aged 18 years and above who consented to take part in the study and have received perceived bad news, including the mentioned diagnoses below by a physician in the last one year. It was planned to include patients with diagnosis including Human Immunodeficiency Virus (HIV) infection, chronic kidney disease, chronic dialysis patients, chronic liver disease, heart failure, diabetes, malignancy, advanced dementia, chronic obstructive pulmonary disease and additional disabling conditions including stroke, Parkinson's disease, asthma, epilepsy and hemophilia.

Physicians working in the Internal Medicine Department during data collection and who were willing to be part were included. Patients who were too ill or too weak to respond and patients with mental disorders preventing the interview were excluded.

**Statistical analysis**: Different statistical analysis methods were used including cross-tabulation between variables and Wilcoxon signed-rank test. Determination of statistical significance using chi-square test was applied using Statistical Package for Social Sciences (SPSS) version 25. All tests with P-Value < 0.05 were considered statistically significant. Before conducting the research, permission was obtained from SPHMMC Institutional Review Board. Informed written consent was obtained from each participant.

## Results

**Patients**: The majority (87.8%) were outpatient. Male responders were 59.2%. The youngest age was 18, and the oldest was 82 with a mean of 46.8. Married individuals were 75.6%, and 27.3% did not have formal education. Unemployed participants were 16.9%, and 59% lived in urban areas (table 1).

**Table 1:** Patient characteristics, SPHMMC, Addis Ababa, 2019

Variables	Frequency	Percent
Patient source		
Outpatient	316	87.8
Ward	44	12.2
Age Group		
18–29	36	10.0
30–39	70	19.4
40–49	111	30.8
50–59	71	19.7
>60	72	20.0
Gender		
Male	203	59.2
Female	140	40.8
Marital status		
Married	260	75.6
Widowed	31	9.0
Divorced	20	5.8
Single	33	9.6
Education level		
Illiterate	98	27.3
Primary school	52	14.5
Secondary school	69	19.2
Certificate or higher	140	39.0
Occupation		
Government employee	121	34.1
Farmer	53	14.9
Private employment	57	16.1
Student	11	3.1
Unemployed	60	16.9
Other	53	14.9
Residence		
Urban	207	59.0
Rural	144	41.0
Site of Disclosure		
At SPHMMC	147	40.8
Another hospital	136	37.7
Health center	53	14.8
Private clinic	24	6.7
Time of Disclosure		
In the last 3 months	138	38.2
3 to 6 months back	75	20.9
6 to 12 months back	147	40.9

The most frequently identified diagnosis was chronic kidney disease 62(17.2%) followed by heart failure 58 (16%), different malignancies 47(13.1%), chronic liver disease and Diabetes each 36(10%). Many (40.8%) had their condition disclosed at SPHMMC, followed by another hospital (37.7%), health center (14.8%) and private clinic (6.7%). Disclosure in the last three months was 38.2% (Table 1).

**Level of performance of SPIKES protocol**: The highest mean percentage response for ‘entirely done’ component was for setting (74.5%) and least was Summary (10.1%) (Table 2).

**Table 2:** Percentage of ‘entirely done’ response to SPIKES protocol components, SPHMMC, Addis Ababa, 2019

SPIKES component	Percentage of ‘entirely done’ response
Setting	74.5%
Perception	51.1%
Invitation	56.3%
Knowledge	15.9%
Empathy	22.3%
Summary	10.1%

**Patient preferences**: The highest response for ‘entirely agree’ was given for ‘physician should check if the message was understood’, ‘should use simple and clear language’ and ‘should give warning’ (Table 3).

**Table 3:** Patient preferences when disclosing bad news, SPHMMC, Addis Ababa, 2019

Question	strongly disagree	Somewhat disagree	very much agree	Entirely/strongly agree
Warn me earlier	2.8	.3	35.6	61.4
Be simple and clear	1.4		36.7	61.9
Check if understood	1.7	.6	35.3	62.5
Ask questions	1.7	.3	38.6	59.4
Summarize and plan	3.1	.3	43.9	52.8
Should ask to bring family	5.6	.6	46.1	47.8
Give an explanation	7.2	.6	44.7	47.5
Know the outcome and prognosis	2.2	.6	43.7	53.5
Same-sex	72.5	3.1	11.1	13.3
Undisturbed atmosphere	1.7	1.9	50.0	46.4
Enough time	.8	1.1	50.3	47.8
Empathic	5.6	5.3	46.4	42.8

**Patient satisfaction**: Only 19.2% were entirely satisfied when asked to grade their satisfaction with their knowledge about the disease and its further management; only 30.6% of the patients were entirely satisfied with the overall interaction with the physician. When ‘entirely satisfied’ response was analyzed, it showed a significant association with whether patients were asked how much information they like to be given (P=0.025).

Comparison of patient desire and report of performance was made using the Wilcoxon signed-rank test. Out of the assessed ten questions, only ‘physicians should ask if patients want a family or friend present’ was not statistically different. The most substantial differences were seen with ‘should use simple and clear language’ (Z= -11.47), ‘should give a chance to ask questions’ (Z= -9.17), ‘should provide summary and plan’ (Z= -11.76), ‘should give detailed explanation’ (Z= -10.16) and ‘should disclose outcome and prognosis’ (Z= -11.68). The questions on ‘should provide warning’ and ‘should be undisturbed atmosphere’ actually was better in reality (Table 4).

**Table 4:** Comparison of Patient preference and actual performance, SPHMMC, Addis Ababa, 2019

Question	Preference	Reality	Test statistics a	

	Patient (‘entirely satisfied’)(%)	(‘entirely satisfied’) (%)	Z	Asymp. Sig. (2-tailed)
Provide warning before	61.4	72.2	-2.711^b^	.007
Simple and clear language	61.9	21.4	-11.471^c^	.000
Give chance to ask questions	59.4	26.2	-9.171^c^	.000
Provide summary and plan	52.8	10.1	-11.769^c^	.000
Ask if you want to bring family or friends	47.8	55.8	-1.942^b^	.052
Give a detailed explanation	47.5	13.6	-10.167^c^	.000
Disclose outcome and prognosis	53.5	11.4	-11.685^c^	.000
Should be undisturbed atmosphere	46.4	59.5	-3.144^b^	.002
Adequate time	47.8	18.7	-8.545^c^	.000
Should be empathetic	42.8	22.3	-5.980^c^	.000

a Wilcoxon signed-rank test

b based on positive ranks

c based on negative ranks

**Physician**: Physicians (111) of all levels of training participated in the study. The majority were males (73%); median age was 28 years, with age ranges from 22 to 55 years. As per the sample size, 61(55%) were internal medicine residents, followed by 22(19.8%) seniors, 4(3.6%) fellows, eight general practitioners (7.2%) and 16 interns (14.4%).

Among the participants, 24(21.6%) completed their undergraduate studies at SPHMMC, followed by the University of Gondar (19.8%) and Jimma University (16.2%) being more common. No sociodemographic characteristics showed any significant association with protocol awareness.

**Awareness and practice of SPIKES**: Among the participants, 87(82.1%) were not aware of the protocol, and 93(83.8%) did not have any specific teaching or training on BBN.

Participants were asked which part of SPIKES they would find the easiest to accomplish; 25.6% said knowledge was the easiest followed by perception (20.6%). Different question asking about the most challenging part revealed setting (28%) and perception (21.6%).

**The attitude of physicians towards SPIKES**: Participants were asked to rate their ability to BBN, and 56.8% said it was fair while 32.4% said it was very good. Fifty-two percent believe that disclosing all information will take away patients' hope and reduce survival. The majority (95.5%) thought that patients want to know their diagnosis and prognosis.

**Emotional response of physicians to BBN**: Half of the participants reported feeling depressed after BBN, while 46.5% feared ending patients' hope (Table 5).

**Table 5:** Questions on the emotional response of physicians, SPHMMC, Addis Ababa, 2019.

Question	Frequency	Percent
Emotions after breaking bad news		
Depressed	78	50.3%
Pity	19	12.3%
Relieved	40	25.8%
Unsafe	7	4.5%
Fearful	6	3.9%
Angry	5	3.2%
Fears in giving bad news		
Being blamed	17	9.1%
Ending hope	87	46.5%
Fear of death	8	4.3%
Fear of own reaction	16	8.6%
Fear of the patient reaction	59	31.6%

## Discussion

Specific components of the protocol were evaluated in different previous studies. Performance of setting-part in this study was 74.5%, similar to a 2018 Polish study, 70.6%; both being significantly higher than 12.5%, a number in a study on schizophrenia patients (4,21). Response to physician being seated was higher in our study compared to the earlier study, 88.6%, and 73.3%, respectively, and eye contact was also higher 94.2% versus 75.7% (4). Having a proper setting including separate room is essential to ensure privacy and comfort, and more needs to be done to provide it as it was shown to be the most valued component (22).

A different response was seen with being asked to bring family with 55.8% in our study compared to only 31.7% in a 2015 Saudi Arabian study (23). This difference could be explained by cultural and religious differences where family is more engaged in communication, and patients call on family to facilitate communication (23,24,25).

Perception performance was 51.1% which is higher compared to 27.7% and 6.3% in two other studies. Similarly, invitation was sought in 56.3% compared to 30.4% and 6.3%.

The two components were also given least importance by patients (4,21,22).

The most significant difference was seen with knowledge showing a mean result of only 15.9% compared to 72.8% (4). This difference can be explained by inclusion of disclosure of prognosis in our study which had positive response of only 11.4%. Patients want their prognosis disclosed in 53.5% of the time, but other studies show more than 89% wanting to be informed (5,7,23). The other knowledge question about use of understandable language was also low (21.4%) and could have resulted from language differences (4,26). On the contrary, assessment in psychiatry, where SPIKES is not extensively evaluated, showed similar rate of 18.8% further highlighting the need for the protocol in non-oncology fields (21).

Emotion part varied significantly, with 22.3% giving an entirely satisfied response compared to 75.3% and 37.5% in others. This isof concern as studies have shown that lack of sympathy can lead to patient dissatisfaction (4,21,27).

The final component, summary, was accomplished in 22.3% compared to 56.9%, which could also potentially be explained by a lack of proper setting (4). It is an important step that reduces uncertainties and presenting treatment options can be a legal requirement (10).

Our patients' desires and priorities are different from other studies. Our patients gave highest value for warning, clear communication and checking if they understood while others, all cancer patients, put setting components, giving detailed and clear information and empathy as most crucial (5,22,25). These differences could be attributed to cultural differences or my be due to our evaluation of medical patients as opposed to cancer patients.

Patient satisfaction is the most widely used indirect measure for evaluating effectiveness of physician-patient communication (28). Patient satisfaction with how bad news was communicated was 30.6%, which is lower compared to the German study that showed satisfaction of 46.2% (5). Other studies have even higher satisfactions with 60%, 88.9% and most patients being satisfied (27,29,30). Previous studies show that satisfaction and patient-physician communication can be affected by communication skills, physician's fear of emotional response and lack of training, among others (1,4,5). This study, however, has shown that asking how much information patients like to be given affects satisfaction.

Patients who were entirely satisfied with the knowledge they were able to attain were only 19.2% compared to 56.9% (4). This is in line with the significant miss-match for a desire for learning in this study.

Among this study, 82.1% of participating physicians are not aware of SPIKES protocol, which was 60% in one study and 27% in medical students (19, 31). However, another 2017 study, in which physicians were asked if they knew any protocol, none were aware of any (17). Eighty-three percent did not have prior training, higher than other studies, where 51.2 % to 70.6% had training (8,10,16,17).

Participants think patients should be informed first in 34.5% of the time, which is similar to a Saudi study of 31.2% and 11% in other studies (15,16). On the other hand, a different study showed preferences of 86.3% (8). This may be influenced by society's cultural practices where family deals with patient affairs instead of direct patient involvement. Cultural influences show variable attitudes in different countries like Saudi Arabia and Korea compared to the United States and other countries(8,10,15,16).

Fifty-two percent of our participants believe disclosure will take away the patient's hopes but only half that (25.5%) in the comparison study (11). Previous studies, including a 2007 systematic review, have shown that disclosure of diagnosis or prognosis does not usually increase patient anxiety. It can actually have benefits including helping patients decide on their subsequent management and can increase hope. On the contrary, withholding information can increase stress on the family and physicians (8,32).

Half of the participants responded that they feel depressed after BBN compared to 40.8% in another study. In addition, 31.6% feared patient's reaction compared to 58.6%. This shows our participants tend to experience more negative emotions like depression (17).

Education and training on communication skills, including how to BBN, can improve physician confidence, ability and decrease negative emotions associated with it, while more than 80% of physicians feel that training should be part of the undergraduate or the residency program (10,16,17,18).

As to the authors' knowledge, there are no large-scale studies done on the subject, particularly in internal medicine. A large number of patients were included in the research, and it was attempted to have full results that incorporate both patient and physician perspective. It serves as a significant starting point for other institutions and other departments to take initiatives on this critical issue.

Both patients and physicians assessed the level of performance of SPIKES themselves with subjective questions, and it may not show the magnitude of the real problem. Objective studies with video recording of interviews with structured and standardized evaluation tools would reveal more accurate results. Many patients started their treatments at other hospitals with different physicians, and results cannot be generalized to SPHMMC. More comprehensive studies at other hospitals should be done to know the magnitude of the problem. The protocol was developed in the United States and is still mainly validated in developed countries. It should be validated in future studies taking into account the cultural and resource limitations available in this country. Finally, most comparisons were between cancer patients, and more studies in other fields should be done.

In conclusion, poor performance concerning the components of the protocol was seen. Patients' desires on how to be told their news is very different from how it is done. Patients are not satisfied with the communication process and with the knowledge they attain during bad news delivery. Physicians should be able to ask what patients want to know as it has effects on their satisfaction.

Most of the participating physicians were not aware of the protocol, nor did they ever receive any training. Many were not comfortable dealing with patients' emotions and did not rate their ability as good. Delivering bad news is a challenging activity and can lead to feelings of depression, fears of ending patients' hopes, and fear of the patients' reaction.

Physicians should be sitting, give clear and detailed explanations including prognosis, in a comfortable environment and end up by summarizing plans of care without fearing patients' social and educational background and have a sympathetic approach while doing so. The belief that disclosure takes away hope is unfounded; it should be abolished and replaced with empathetic communication. However, not all responsibility should be left to the physicians. Finding proper settings was identified as a significant impediment, and the entire healthcare system should be organized, recognizing patients' needs for a conducive atmosphere for discussion. This should be the responsibility of healthcare institutions, hospital administrators, quality assurance offices, healthcare consultants and significant regulators like the Ministry of Health. Undergraduate medical schools, along with fellowship programs, should be designed in a way to prepare their students starting early on what a proper way of communication is and incorporate SPIKES into their studies. Practical demonstrations and other skill-building exercises should be part of routine teaching process. Strict follow-up schemes, and regular updates should be provided as needed. This should be applied by teachers, curriculum developers and accreditation bodies.
